# Phenotypic diversity within a *Pseudomonas aeruginosa* population infecting an adult with cystic fibrosis

**DOI:** 10.1038/srep10932

**Published:** 2015-06-05

**Authors:** Shawn T. Clark, Julio Diaz Caballero, Mary Cheang, Bryan Coburn, Pauline W. Wang, Sylva L. Donaldson, Yu Zhang, Mingyao Liu, Shaf Keshavjee, Yvonne C.W. Yau, Valerie J. Waters, D. Elizabeth Tullis, David S. Guttman, David M. Hwang

**Affiliations:** 1 Department of Laboratory Medicine and Pathobiology, University of Toronto, Toronto, Canada; 2 Latner Thoracic Surgery Research Laboratories, University Health Network, Toronto, Canada; 3 Department of Cell & Systems Biology, University of Toronto, Toronto, Canada; 4 Centre for the Analysis of Genome Evolution & Function, University of Toronto, Toronto, Canada; 5 Department of Pediatric Laboratory Medicine, Division of Microbiology, The Hospital for Sick Children, Toronto, Canada; 6 Department of Pediatrics, Division of Infectious Diseases, The Hospital for Sick Children, Toronto, Canada; 7 Department of Medicine, Division of Respirology, St. Michael’s Hospital, Toronto, Canada; 8 Laboratory Medicine Program, University Health Network, Toronto, Canada

## Abstract

Chronic airway infections caused by *Pseudomonas aeruginosa* contribute to the progression of pulmonary disease in individuals with cystic fibrosis (CF). In the setting of CF, within-patient adaptation of a *P. aeruginosa* strain generates phenotypic diversity that can complicate microbiological analysis of patient samples. We investigated within- and between- sample diversity of 34 phenotypes among 235 *P. aeruginosa* isolates cultured from sputum samples collected from a single CF patient over the span of one year, and assessed colony morphology as a screening tool for predicting phenotypes, including antimicrobial susceptibilities. We identified 15 distinct colony morphotypes that varied significantly in abundance both within and between sputum samples. Substantial within sample phenotypic heterogeneity was also noted in other phenotypes, with morphotypes being unreliable predictors of antimicrobial susceptibility and other phenotypes. Emergence of isolates with reduced susceptibility to β-lactams was observed during periods of clinical therapy with aztreonam. Our findings confirm that the *P. aeruginosa* population in chronic CF lung infections is highly dynamic, and that intra-sample phenotypic diversity is underestimated if only one or few colonies are analyzed per sample.

Cystic fibrosis (CF) is a fatal genetic disease that predisposes patients to polymicrobial infection of the lungs^1^,^2^. Chronic infections caused by *Pseudomonas aeruginosa* are associated with increased severity of lung disease and premature death^3^,^4^.

In the diseased CF lung, *P. aeruginosa* is exposed to a range of selective pressures including host immune responses, competing organisms and antimicrobials, which are thought to drive genetic and phenotypic diversity within an infecting strain over time^1^,^5^,^6^,^7^. Various airway-specific adaptations are postulated to favour persistence and lead to host-tolerant clonal lineages that are less cytotoxic, better at evading the immune system, more resistant to antimicrobials and less metabolically active than their ancestral strains^5^,^8^,^9^,^10^,^11^,^12^,^13^. These adaptations correlate with increasingly diverse colony morphologies, or morphotypes, that are visible during routine culture of expectorated sputum^12^,^14^,^15^.

Conventional approaches to characterizing *P. aeruginosa* cultured from expectorated sputum and bronchoalveolar lavage fluid in CF have relied on sampling single or few colonies per specimen for further phenotypic analysis, often based on morphotype abundance^11^,^14^,^16^. Previous studies of CF-evolved phenotypes have either been cross-sectional, profiled small numbers of isolates from multiple patients, focused largely on specific phenotypes or on highly transmissible epidemic strains such as the Liverpool (LES) or Prairie (PES) strains^5^,^10^,^16^,^17^,^18^,^19^,^20^,^21^,^22^,^23^. The few studies to date with increased sampling depth have found discordant relationships between morphotype and other phenotypic characteristics when multiple isolates from the same sample are tested, with colonies of the same morphotype often displaying different antimicrobial susceptibility profiles^6^,^14^,^24^,^25^,^26^. This variability provides evidence to fuel concerns over the reliability of using morphotype-based approaches to predict additional phenotypes of clinical significance in *P. aeruginosa* isolates from the CF airways.

The objectives of the present study were (i) to characterize within-host diversity of morphotypes and adaptive phenotypes in a non-LES or PES strain of *P. aeruginosa*; (ii) to examine temporal changes in patterns of susceptibility following exposure to antimicrobials, and (iii) to identify relationships between morphotypes and other phenotypic characteristics, particularly antimicrobial susceptibility.

## Results

### Diversity of P. aeruginosa colony morphologies within and between samples

**Figure 1:**
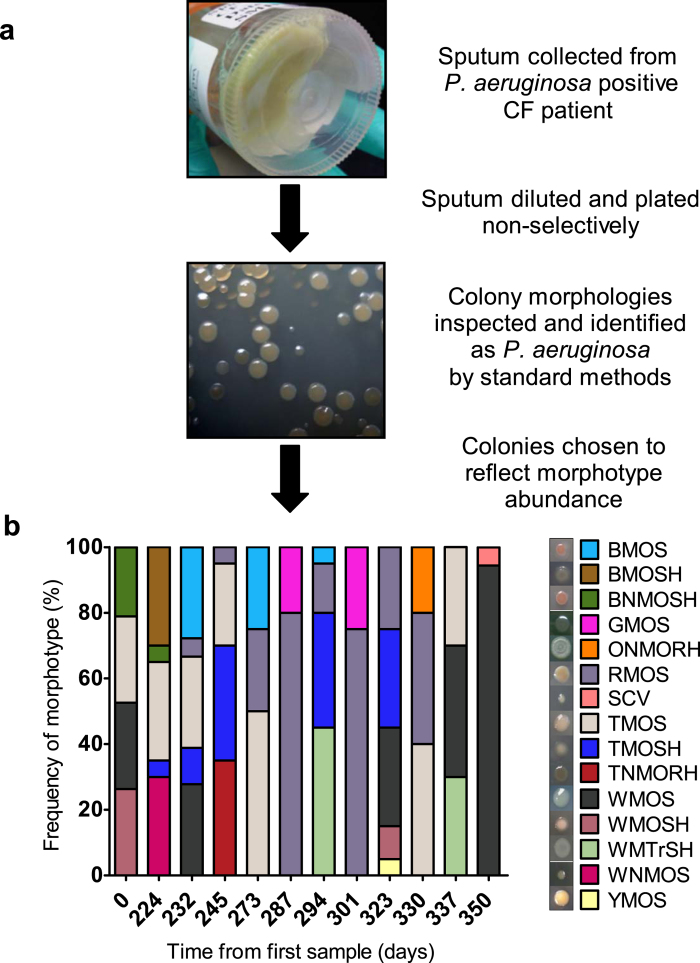
Temporal diversity of *P. aeruginosa* colony morphologies found in the sputum of a CF patient. Diversity was assessed through (**A**) culture-dependent identification and (**B**) characterization of colony morphologies by morphotype over 350 days.

Twelve sputum samples were collected over a 350-day period from patient CF67, a 34 year old female (ΔF508/CFTRdele2,3 genotype) with advanced lung disease (average forced expiratory volume in 1 second (FEV_1_) of 21% predicted; range: 15 to 25%). Due to her poor baseline lung function and lack of recovery between antibiotic courses, the patient did not meet criteria for exacerbation episodes during the study period. Phenotypic analyses were performed on 235 isolates which passed quality control (range: 18-20 isolates per specimen), all of which were of the same multilocus sequence type (ST), ST-274 (DSG, unpublished data). Fifteen distinct *P. aeruginosa* morphotypes were evident based on differences in 8 morphologic features (Supplementary Table 1), with a mean of 4 morphotypes per sputum sample (range: 2–5) (Fig. 1). Ninety percent of isolates were mucoid. The overall abundance of each morphotype fluctuated over time, without any single morphotype predominating in all samples (Fig. 1). Nine morphotypes were present in more than 1 sample, with no discernable trend in morphotype abundance over time.

### Diversity of chronic infection and virulence-associated adaptive phenotypes

**Figure 2:**
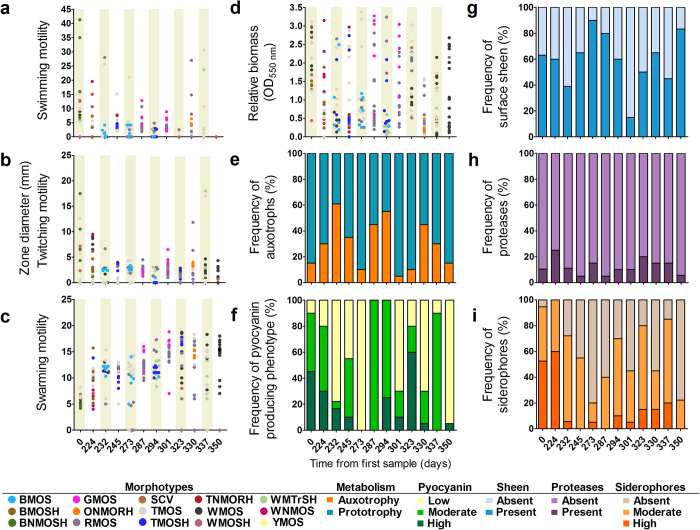
Diversity of adaptive phenotypes. Fluctuations in motility-associated phenotypes (A-**D**) are represented by individually colour-coded morphotype, while those observed for amino acid auxotrophy (**E**), pyocyanin production (**F**), iridescent surface sheen (**G**), protease production (**H**), and siderophore production (I), are depicted at the population level. Phenotypes were measured among twenty isolates from each sputum sample with the exception of days 0 (19 isolates), 232 (18 isolates) and 350 (18 isolates). Each data point in panels A-C represents the mean of three replicates and six replicates in panel D, with standard deviation removed for presentation.

Substantial variability was found in chronic infection and virulence-associated phenotypes from isolates collected both within single sputum samples and between different samples (Fig. 2). No clear temporal trends were apparent, although earlier isolates typically demonstrated higher twitching and swimming motilities and lower swarming motility compared to isolates from later samples (Fig. 2A–C). Within any given sample, wide gradients were observed in the ability of isolates to produce biofilm (Fig. 2D). Similarly, a significant minority of isolates had auxotrophic metabolism (range: 5% to 61% of isolates within a sample), with the most prevalent auxotrophies being for aspartate, glutamate, histidine, leucine, methionine and proline (Fig. 2E and S1). Comparable fluctuations in the frequencies of secreted virulence factors were also found between samples (Fig. 2F–I).

Despite the diversity of phenotypes, several relationships between phenotypic traits emerged (Supplementary Figure 2). Swimming and twitching motilities were modestly correlated (Spearman *r* = 0.49, *p* < 0.0001) and were both weakly negatively correlated with swarming (Spearman *r* = −0.215, *p* = 0.0009). Expression of several phenotypes associated with earlier stages of infection^16^ were also moderately correlated, for example, of siderophores with pyocyanin (Spearman *r* = 0.43, *p* < 0.0001) or with proteases (Spearman *r* = 0.44, *p* < 0.0001). In contrast, these virulence-associated traits were commonly negatively correlated with chronic infection or persistence-related phenotypes, although not uniformly (Supplementary Figure 2).

### Within- and between-sample variation in antimicrobial susceptibility

**Figure 3:**
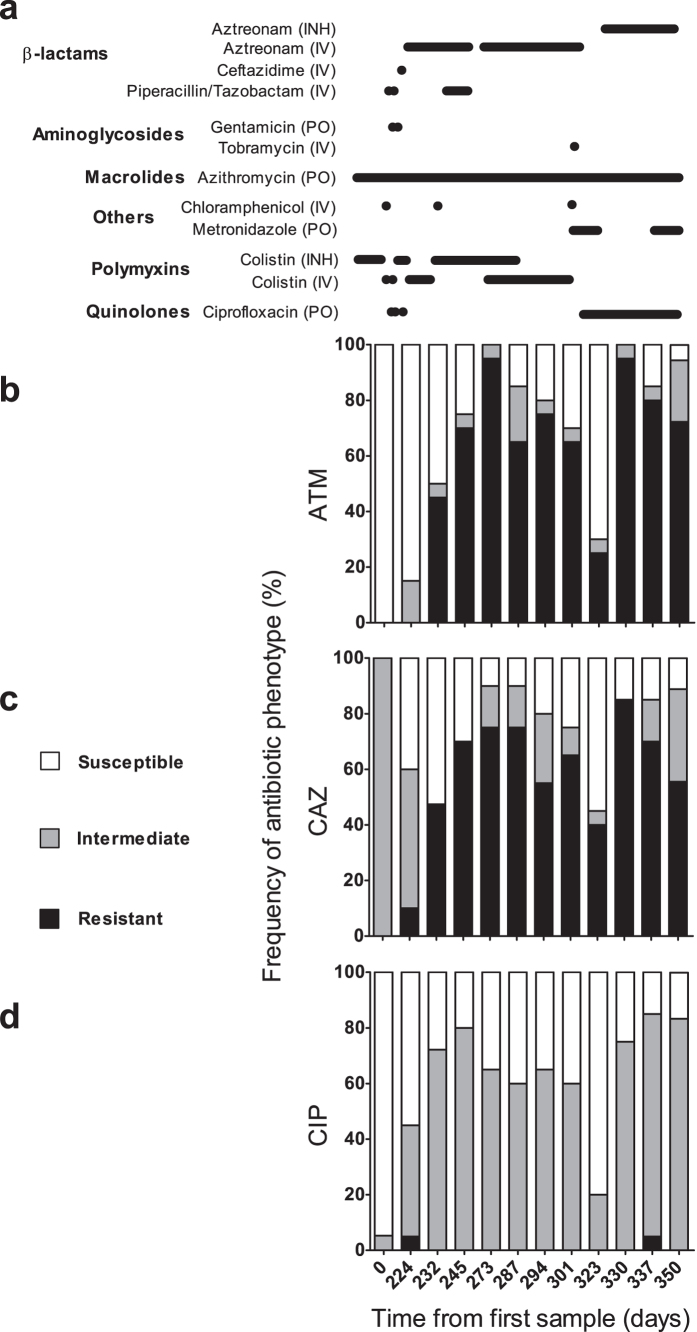
Variation in antimicrobial susceptibility patterns over time. Changes in (**A**) antibiotic exposure and *in vitro* responses to (**B**) aztreonam, (**C**) ceftazidime, and (**D**) ciprofloxacin. Susceptibilities are expressed as the percentage of isolates recovered from a given sample with a particular response phenotype and defined as susceptible (MIC ≤ 8 μg/mL for ATM, CAZ; ≤ 1 μg/mL for CIP), intermediate (MIC of 16 μg/mL for ATM, CAZ; 2 μg/mL for CIP) or resistant (MIC ≥ 32 μg/mL for ATM, CAZ; ≥ 4 μg/mL for CIP) by CLSI interpretive criteria. Delivery routes for antimicrobial therapies are defined as inhaled (INH), parenteral (IV) or oral (PO).

Longitudinal and cross-sectional variations in susceptibility to anti-pseudomonal antibiotics were assessed by agar dilution. All isolates were susceptible to amikacin and tobramycin and resistant to imipenem, with the exception of a single isolate collected on day 330. Considerable within- and between-sample diversity in susceptibility to aztreonam and ceftadizime was observed (Fig. 3), with 16 susceptibility profiles identified for these two antibiotics (mean: 6 profiles per sputum sample; range: 1 to 10, Supplementary Table 2).

Following the administration of parenteral or inhaled aztreonam therapy on days 219, 263 and 325, increased frequencies of isolates resistant to either or both aztreonam and ceftazidime were found when comparing MICs from samples collected on days 224 versus 232 (*p* = 0.003, *p* = 0.04 for aztreonam and ceftazidime respectively), and days 323 versus 330 (*p* < 0.0001, *p* = 0.02 for aztreonam and ceftazidime respectively; Fig. 3; Supplementary Table 3). Resistance to aztreonam and ceftazidime were moderately correlated (*r* = 0.52, *p* < 0.0001, Supplementary Figure 2). A substantial decrease in MICs for both aztreonam and ceftazidime was noted among isolates cultured from the day 323 sample, which was collected following the discontinuation of aztreonam therapy on day 311 (Fig. 3; Supplementary Table 3). Reduced susceptibility to ciprofloxacin was also noted in some isolates, in parallel with that of aztreonam and ceftazidime (Fig. 3). Thus, although no temporal trends in morphotypes were evident, antimicrobial resistance increased over time following administration of anti-pseudomonal antibiotics.

### Colony morphotypes as predictors of other phenotypes

Co-occurrences of colony morphologies and other phenotypes were analyzed to identify potential associations (summarized in Supplementary Tables 4 and 5). In most cases, morphotypes lacked strong associations with specific phenotypic characteristics, with significant heterogeneity in phenotypic profiles among isolates of the same morphotype (Supplementary Tables 4 and 5). The few associations that were observed included heavy production of exopolysaccharide and reduced susceptibilities to aztreonam and/or ceftazidime by several mucoid morphotype groups, and the induction of moderate pyocyanin production by the white, mucoid, translucent, smooth with outer halo morphotype (WMTrSH) group on cetrimide agar.

**Figure 4:**
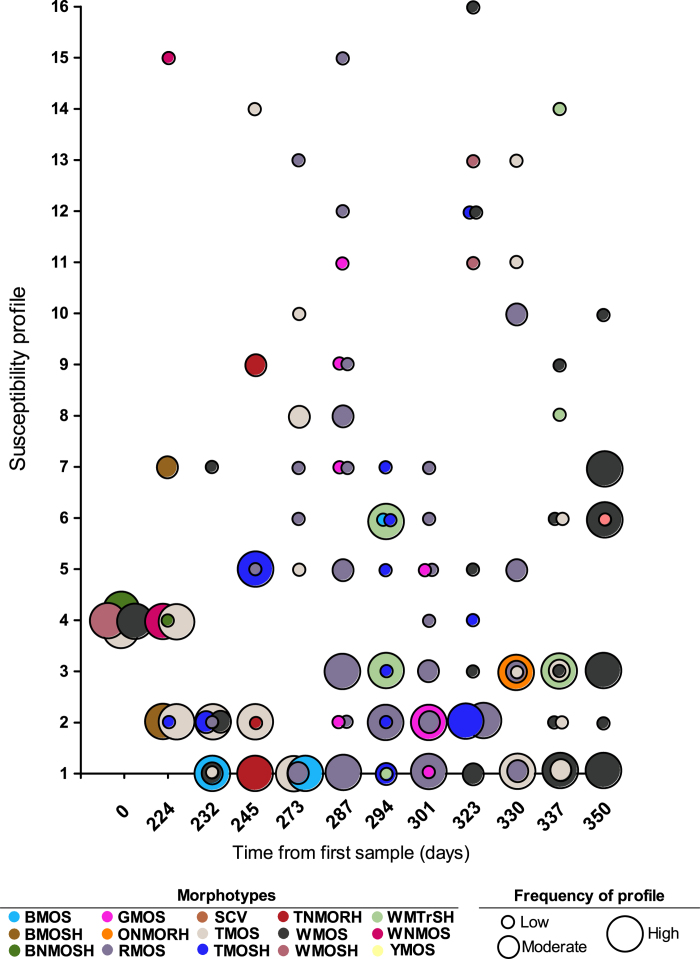
Variation in β-lactam susceptibility within and between morphotypes. The frequency of a particular antimicrobial susceptibility profile (for the antibiotics aztreonam and ceftazidime) for any morphotype is depicted by the size of the corresponding circle, representing low (single isolate with the susceptibility profile), moderate (2–3 isolates with the susceptibility profile) or high ( ≥ 4 isolates with the susceptibility profile) frequencies.

Overall, colony morphology was poorly predictive of phenotypic properties including antimicrobial resistance as determined by multivariate analysis (Table 1). Only two morphotypes, WMTrSH and brown, mucoid, opaque and smooth (BMOS) were predictive of antimicrobial resistance, with WMTrSH more likely to be resistant to aztreonam and less likely to be resistant to ceftazidime, and BMOS more likely to be resistant to ceftazidime. Apart from these two low- to moderate-abundance morphotypes (together comprising 26 of 235 isolates), colony morphotypes were unreliable predictors of antimicrobial susceptibility, with a mean of 5 susceptibility profiles reported per morphotype (range: 1–13) for aztreonam and ceftazidime alone (Supplementary Table 2), and with a high degree of variation between isolates of the same morphotype, both within single sputum samples and between samples collected at different times (Fig. 4).

## Discussion

In the present study, we have described the most extensive longitudinal analysis to date of morphotypic and phenotypic diversity in a *P. aeruginosa* strain from an adult with CF. In agreement with previous work on LES and PES in CF adults^6^,^10^,^24^, significant within- and between-sample diversity was also identified among *P. aeruginosa* isolates of sequence type ST-274 in our study. This diversity was expansive and in some instances, we observed the co-occurrence of phenotypes typically considered markers of chronic adaptation, such as an iridescent surface sheen, mucoidy, or less virulence factor production^8^,^12^,^13^,^16^,^27^,^28^, with traditional wild-type phenotypes such as active twitching motility and biofilm formation^16^,^27^ within single isolates. Extensive within-sample diversity was also noted in antimicrobial susceptibility profiles similar to recent studies that have tested more than 20 isolates per sample or morphotype^6^,^24^,^25^,^26^. This was particularly evident for the anti-pseudomonal β-lactams aztreonam and ceftazidime; however, a clear trend of increased resistance was apparent over time.

There was substantial sample-to-sample variability in the composition of morphotypes cultured from patient sputum, without evidence of persistent morphotypes or temporal trends in their abundance. In agreement with a previous report^14^, we found that morphotype is not a sufficiently reliable predictor of other phenotypes to be used as a criterion for the selection of colonies for further analysis, especially for antimicrobial susceptibility testing. Notably, the phenotypic diversity captured by our study arose within a short period of time, with all 235 isolates sharing a most recent common ancestor approximately 3 years prior to the last sputum sample based on preliminary genome analyses (Hwang & Guttman, unpublished data). This indicates that this phenotypic diversification occurred rapidly and the phenotypes that diversified largely did so independently.

In contrast to earlier studies^6^,^24^, we observed rapid shifts in antimicrobial susceptibility profiles in as few as 7 days that appeared to be temporally related to antibiotic administration. The findings are consistent with models of adaptive resistance^29^,^30^, and are suggestive of significant fitness costs, defined as a detrimental impact on the ability of an organism to survive and propagate in a particular environment, associated with maintaining resistance in the absence of selection by a specific antimicrobial agent. Similarly, it is likely that determinants of antimicrobial resistance are not uniform across members of an established *P. aeruginosa* population within the CF lung, and imply the presence of a reservoir of resistance determinants contributing to differential resistance phenotypes that are selected under specific environmental conditions^31^.

The precise microenvironmental pressures driving diversification of other phenotypic traits within *P. aeruginosa* populations in the CF lung may prove more enigmatic than for antimicrobial resistance, but a similar model seems likely to apply. With the costs of DNA sequencing rapidly decreasing, whole genome sequencing of large numbers of isolates coupled with phenotypic characterization and genome-wide association analyses is a promising approach to identifying specific genetic determinants contributing to such diversity^32^,^33^.

Potential limitations of this work include the use of expectorated sputum, which makes it impossible to determine the location(s) in the lungs from which the samples/isolates originated. Uncertainty in the spatial distribution of bacterial populations is however unavoidable for a longitudinal study of this nature. While direct sampling from lung compartments by bronchoalveolar lavage may permit for more accurate localization, samples could not be collected frequently, given its invasive nature. Similarly, analyzing explanted lungs^2^,^34^ would only allow for a single snapshot of spatial distribution at the time of transplantation or death, and provide limited information regarding population dynamics over the course of disease progression. Due to the relatively recent ancestry of these isolates, we would anticipate that the study population would be phenotypically and genotypically homogeneous, which would bias our analysis towards false-positive correlations. However, despite this population structure, we observed very few correlated phenotypes and little association between morphotype and antimicrobial susceptibility. The absence of clear phenotype-phenotype association in this population strengthens the support for a model of rapid independent phenotypic adaptation, indicating that the use of morphotypes as a surrogate for other phenotypes may obscure important trends, even over very short time intervals (*i.e.* from sample-to-sample).

In summary, our data confirm the presence of tremendous phenotypic heterogeneity within a non-LES or PES *P. aeruginosa* population chronically infecting the CF lung. These populations are significantly more complex and dynamic than can be described by the analysis of any single isolate and can fluctuate rapidly to changing selective pressures. Furthermore, colony morphotypes are poor predictors of other phenotypic characteristics including antimicrobial susceptibility, even among isolates from the same sputum sample. Colony morphology should therefore be used with caution as a screening tool for selecting isolates for antimicrobial susceptibility testing in the setting of chronic CF lung infections.

## Materials & methods

### Specimen collection and microbiological analysis

Protocols for specimen collection and use were approved by the Research Ethics Boards of St. Michael’s Hospital (protocol #09-289) and the University Health Network (protocol #09-0420-T), and informed consent for specimen collection and use under these protocols was obtained from the study subject (CF67). All experiments were conducted in accordance with the *Tri-Council Policy Statement: Ethical Conduct for Research Involving Humans*, of the Canadian Institutes of Health Research (CIHR), the Natural Sciences and Engineering Research Council of Canada (NSERC), and the Social Sciences and Humanities Research Council of Canada (SSHRC).

Twelve sputa were collected over the span of 350 days between November 2010 (day 0) and November 2011 (day 350) from an adult female CF patient known to be chronically infected with *P. aeruginosa* for at least 12 years and followed at St. Michael’s Hospital CF clinic (Toronto, ON, Canada). Sputa were transported to the laboratory on ice, solubilized by homogenization with Sputolysin® (EMD Millipore, CA, USA), serially diluted (10^−3^ to 10^−5^) in sterile water and plated in triplicate onto Luria-Bertani (LB) agar (Wisent Inc., QC, Canada) without antibiotics. Cultures were grown aerobically for up to 72 h at 37 °C. Colonies were classified into morphotypes by visual inspection by a single observer and described using 8 identifiers of physical appearance (pigmentation, size, surface texture, surface sheen, opacity, mucoidy, autolysis and margin shape). We defined a morphotype as a unique combination of these physical features, with size being used to distinguish small colony variants (SCV) (Table S1). Twenty *P. aeruginosa* colonies were selected per sample, with proportions of different morphotypes chosen relative to their overall abundance in the sputum culture as described by Mowat *et al.* (2011)^6^. Isolates were cryopreserved at −80 °C in 20% (v/v) glycerol after a single subculture in LB broth (Wisent Inc., QC, CA) and confirmed as *P. aeruginosa* biochemically by oxidase testing and a secondary subculture onto MacConkey and cetrimide agars (Becton Dickinson, MD, USA).

### Phenotypic characterization of *P. aeruginosa* isolates

All phenotypic assays (described below) were performed by a single experimenter as three independent biological replicates unless otherwise specified. The *P. aeruginosa* strains PAO1 and PAK were used as positive controls to assay motility, biofilm formation and secreted virulence factors, and negative controls for mucoidy. The *P. aeruginosa* ATCC 27853 and *E. coli* ATCC 25922 strains were used for antimicrobial susceptibility testing quality control.

### Motility and biofilm formation

Subsurface twitching and swimming assays were performed as described^35^ with modification. Briefly, twitching was measured by stab-inoculating individual colonies to the bottom of thin 1.5% (wt/vol) LB agar plates. Twitch zones were stained with 1% (wt/vol) crystal violet after 72 h of incubation (37 °C for 48 h, 25 °C for 24 h). For swimming, colonies were stab-inoculated into 0.3% (wt/vol) LB agar plates and incubated for 24 h at 37 °C. Swarming was assessed by surface inoculating an aliquot of stationary phase liquid culture onto 0.5% (wt/vol) BM2 agar supplemented with 0.5% casamino acids^36^ and grown for 48 h at 37 °C. All motility zone diameters were measured in millimetres.

Biofilm formation was assayed as described by Merritt *et al.* (2011)^37^. Briefly, stationary-phase cultures were diluted 1:100 in fresh LB medium, seeded into 6 replicate wells of a 96 well non-tissue culture treated microtitre plate and incubated in static culture for 24 h at 37 °C. Crystal violet-stained biomass, 0.1% (vol/vol), was quantified by measuring the optical density at 550 nm.

### Secreted virulence factors and metabolism

Pyocyanin production was assessed qualitatively by visual inspection of cultures grown on Cetrimide agar (Becton, Dickinson, MD, USA). Production was classified as low (+), or high (++), and isolates that exhibited growth but lacked pyocyanin production were scored as (+/−).

Extracellular proteases and siderophores were assessed in a similar manner by examining clearing zones produced on either skim milk (Becton Dickinson, MD, USA), or chrome azurol S agar^38^, respectively. Isolates lacking either trait were classified as negative (−), while those with a moderate diameter (+) or a large diameter (++) were scored accordingly.

Amino acid auxotrophy was screened by growth on M9 minimal salts agar (Sigma-Aldrich, ON, Canada) supplemented with 20% (wt/vol) glucose. Auxotrophic isolates were identified by a lack of growth following 48–72 h of incubation at 37 °C. The identification of auxotrophies for specific amino acids was characterized as described earlier, with the addition of one of 18 amino acids to the agar at physiologically relevant concentrations^39^.

Mucoidy was assessed visually following 48 h incubation on LB and yeast extract mannitol (YEM) agar. Growth was scored as as non-mucoid (−), lightly mucoid (+), moderately mucoid (++), or heavily mucoid (+++), which was noted by the transfer of the exopolysaccharide alginate onto the opposing lid of the Petri dish^40^.

### Antimicrobial susceptibility testing

Each *P. aeruginosa* isolate was screened for antimicrobial susceptibility by the agar dilution method in accordance with Clinical and Laboratory Standards Institute (CLSI) procedures^41^. Susceptibility profiles were determined for the anti-pseudomonal β-lactams (aztreonam, ceftazidime, imipenem), fluoroquinolones (ciprofloxacin) and aminoglycosides (amikacin, tobramycin) (Sigma Aldrich, ON, Canada). A two-fold dilution series was chosen for each antibiotic to reflect the *P. aeruginosa* interpretive breakpoints^41^. Growth was assessed following 24 and 48 h of incubation on Mueller-Hinton agar (Becton, Dickinson, MD, USA), without prior knowledge of clinical metadata. The minimum inhibitory concentration (MIC), the highest concentration of each antibiotic to completely inhibit the growth of an isolate, was reported as the median of three independent experiments. For the β-lactam antibiotics aztreonam and ceftazidime, susceptibility profiles were identified as each MIC combination reported among isolates.

### Statistical analysis

All statistical analyses were performed in SAS 9.3 (SAS Institute Inc. Cary, NC, USA). Phenotypes were treated as either categorical or continuous variables and analyzed as appropriate. Associations between morphotype and categorical phenotypes were tested by chi- square analysis, while associations between morphotype and continuous phenotypes were tested by Welch’s *t*-test. Only the most abundant morphotypes (n ≥ 10) were included in the analysis. A Bonferroni correction was applied to adjust for multiple phenotypic comparisons in the bivariate analysis (*p* ≤ 0.004) for *p* values to be considered significant with 95% confidence. Variations in MIC between samples dates were determined using a two-tailed *t*-test (*p* ≤ 0.05). Correlations between phenotypes were determined by calculating the Spearman correlation coefficient and visualizing potential associations as a heatmap using the gplots package in R 3.0.1 (R Development Core Team, 2014. http://www.R-project.org). The strength of phenotypic predictors was estimated by odds ratio using multivariate logistic regression, with all independent predictors being identified by stepwise elimination and considered significant on a 95% confidence interval at *p* ≤ 0.05.

## Additional Information

**How to cite this article**: Clark, S. T. *et al.* Phenotypic diversity within a *Pseudomonas aeruginosa* population infecting an adult with cystic fibrosis. *Sci. Rep.* **5**, 10932; doi: 10.1038/srep10932 (2015).

## Supplementary Material

Supplementary Information

## Figures and Tables

**Table 1:** Phenotypes predicted by colony morphotype.

***P. aeruginosa*** **morphotype**^a^						
**Phenotype predictors**	**BMOS**	**RMOS**	**TMOS**	**TMOSH**	**WMOS**	**WMTrSH**
**Motility–related**						
Biofilm formation	–	–	–	–	–	0.14 (0.02–0.85)
Swimming	–	–	–	–	0.86 (0.76–0.98)	–
Twitching	–	–	–	0.61 (0.76–0.98)	–	–
						
**Antimicrobial susceptibility**						
Aztreonam resistance	–	–	–	–	–	4.4 (1.6–12.3)
Ceftazidime resistance	5.9 (1.6–21.6)	–	–	–	–	0.29 (0.12–0.71)
						
**Virulence–associated**						
EPS production	–	2.2 (1.2–4.1)	3.0 (1.6–6.4)	–	–	–
Surface sheen	0.14 (0.03–0.56)	2.5 (1.1–5.7)	–	–	–	–
Pyocyanin production	–	–	–	3.0 (1.6–5.8)	–	–
Siderophore production	–	–	–	–	–	0.20 (0.05–0.74)

^a^presented as odds ratio with 95% confidence interval.
