# Supplementary material for: Phenotypic diversity within a Pseudomonas aeruginosa population infecting an adult with cystic fibrosis
Source: Sci Rep. 2015 Jun 5;5:10932. doi: 10.1038/srep10932 (PMC4456944; doi:10.1038/srep10932)
Supplement: Supplementary Information [file srep10932-s1.pdf]

## Supplementary Information

Phenotypic diversity within a *Pseudomonas aeruginosa* population infecting an adult with cystic fibrosis

Shawn T. Clark, Julio Diaz Caballero, Mary Cheang, Bryan Coburn, Pauline W. Wang, Sylva L. Donaldson, Yu Zhang, Mingyao Liu, Shaf Keshavjee, Yvonne Y.C. Yau, Valerie J. Waters, D. Elizabeth Tullis, David S. Guttman and David M. Hwang[#]

[#] **Corresponding author**: Email: David.Hwang@uhn.ca

This supplementary PDF file includes:

Figures S1 to S2
Tables S1 to S5

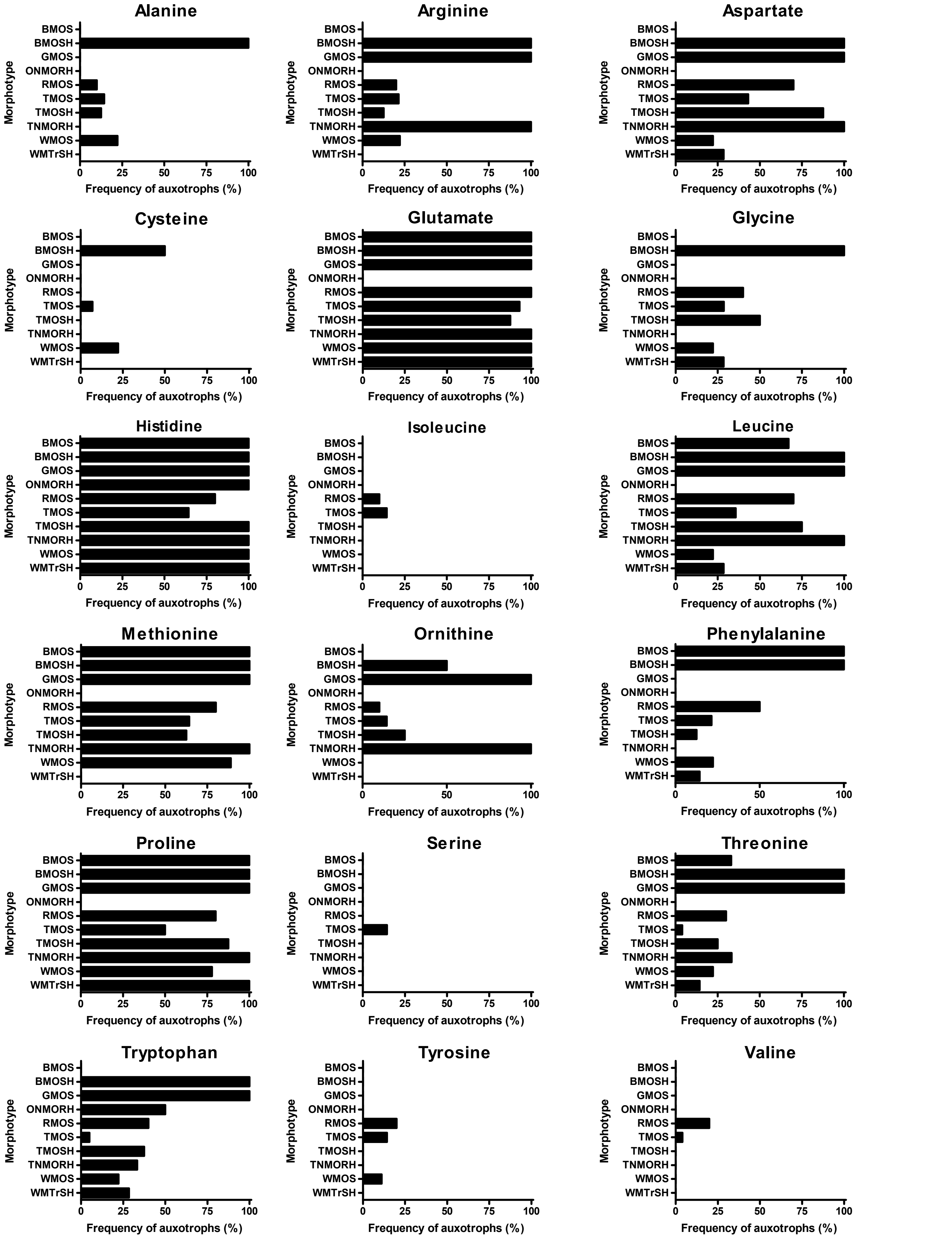


Figure S1. Morphotype-specific amino acid auxotrophies found among the 235 *P. aeruginosa* isolates from patient CF67. Frequencies for each type of auxotrophy are expressed as the proportion of the total number of isolates with a given morphotype that required supplementation of the amino acid for growth in M9 minimal salts agar

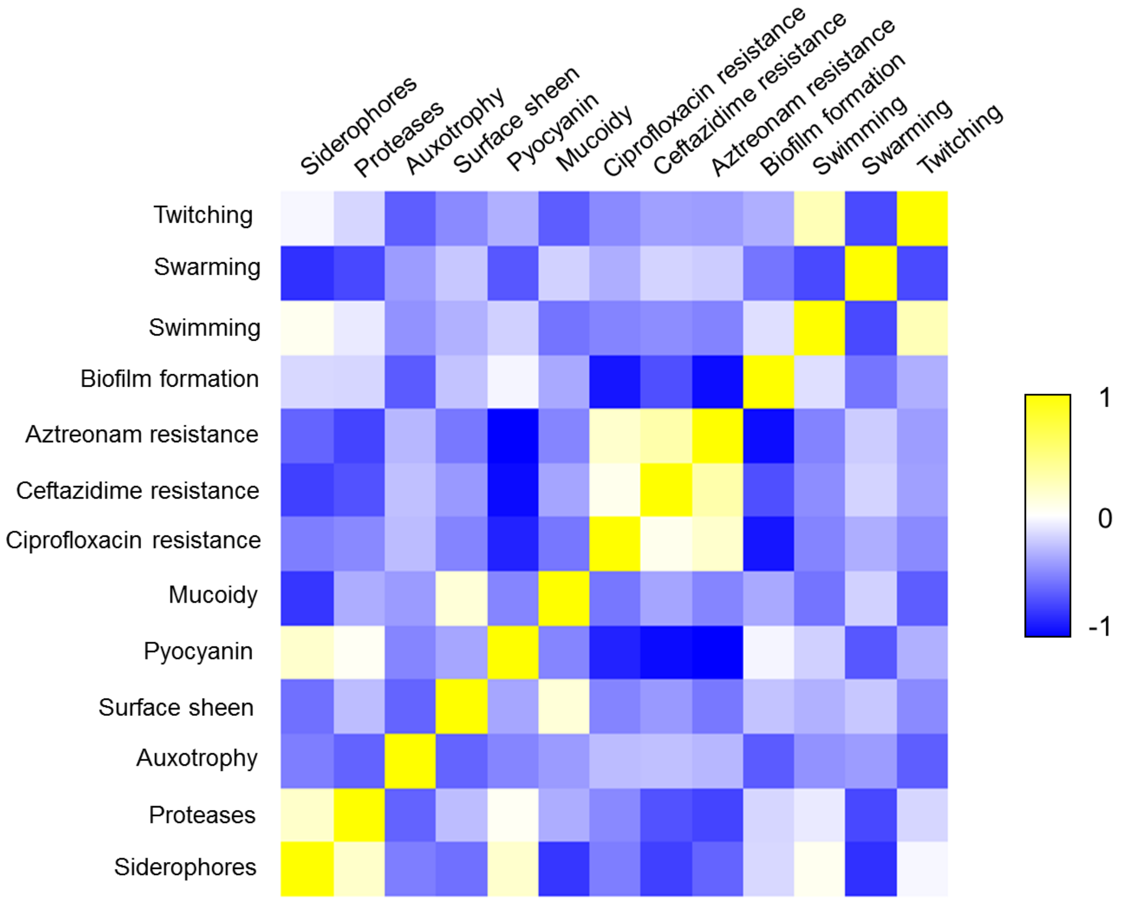


Figure S2. Correlation matrix of phenotype-phenotype associations as determined by Spearman rank correlation coefficient. Yellow indicates strong positive correlation between any given phenotype pair (Spearman rank cofficient closer to 1) while blue indicates strong inverse correlation (Spearman rank coefficient closer to -1) and white indicates no correlation between phenotype pairs (0).

Table S1. Descriptions of *P. aeruginosa* colony morphologies cultured from the sputum of patient CF67

| | Pigmentation | | | | | | | Mucoid | Opacity | Surface texture | | | | Margin | Descriptor[b] | Number of isolates |
|---|---|---|---|---|---|---|---|---|---|---|---|---|---|---|---|---|
| | Brown | Green | Orange | Red | Tan | White | Yellow | | | Smooth | Rough | Halo[a] | Autolysis | Round | | |
| 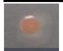 | + | - | - | - | - | - | - | + | + | + | - | - | - | + | BMOS | 11 |
| 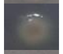 | + | - | - | - | - | - | - | + | + | + | - | + | - | + | BMOSH | 6 |
| 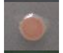 | + | - | - | - | - | - | - | - | + | + | - | + | - | + | BNMOSH | 5 |
| 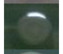 | - | + | - | - | - | - | - | + | + | + | - | - | - | + | GMOS | 9 |
| 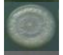 | - | - | + | - | - | - | - | - | + | - | + | + | + | + | ONMORH | 4 |
| 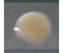 | - | - | - | + | - | - | - | + | + | + | - | - | - | + | RMOS | 54 |
| 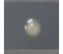 | - | - | - | - | + | - | - | - | + | + | - | - | - | + | SCV | 1 |
| 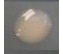 | - | - | - | - | + | - | - | + | + | + | - | - | - | + | TMOS | 45 |
| 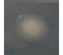 | - | - | - | - | + | - | - | + | + | + | - | + | - | + | TMOSH | 23 |
| 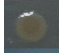 | - | - | - | - | + | - | - | - | + | + | + | + | - | + | TNMORH | 7 |
| 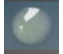 | - | - | - | - | - | + | - | + | + | + | - | - | - | + | WMOS | 41 |
| 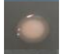 | - | - | - | - | - | + | - | + | + | + | - | + | - | + | WMOSH | 7 |
| 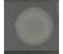 | - | - | - | - | - | + | - | + | - | + | - | + | - | + | WMTrSH | 15 |
| 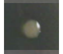 | - | - | - | - | - | + | - | - | + | + | - | - | - | + | WNMOS | 6 |
| 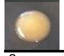 | - | - | - | - | - | - | + | + | + | + | - | - | - | + | YMOS | 1 |

[a] Feature was identified on mucoid colonies as a transparent EPS layer surrounding a central opaque colony and in non-mucoid variants as a non-concentric outer ring

[b] Morphotype descriptors are acronyms that describe the colony type and follow the naming order of colour, mucoidy, opacity, texture and any additional distinguishing surface properties

Table S2. Temporal variation and diversity of antibiograms for the β-lactam antibiotics aztreonam and ceftazidime

| Profile[a] | Susceptibility (μg/mL)[b] | | Time from first sample (days)[c] | | | | | | | | | | | |
|---|---|---|---|---|---|---|---|---|---|---|---|---|---|---|
| | ATM | CAZ | 0 | 224 | 232 | 245 | 273 | 287 | 294 | 301 | 323 | 330 | 337 | 350 |
| 1 | | | - | - | +++ | +++ | +++ | +++ | +++ | +++ | ++ | +++ | +++ | +++ |
| 2 | | | - | +++ | +++ | +++ | - | ++ | +++ | +++ | +++ | - | ++ | + |
| 3 | | | - | - | - | - | - | +++ | +++ | ++ | + | +++ | +++ | - |
| 4 | | | +++ | +++ | - | - | - | - | - | + | + | - | - | - |
| 5 | | | - | ++ | + | - | + | ++ | + | + | - | - | - | +++ |
| 6 | | | - | - | - | - | + | - | +++ | + | - | - | ++ | +++ |
| 7 | | | - | - | - | +++ | + | ++ | + | ++ | + | ++ | - | +++ |
| 8 | | | - | - | - | - | ++ | ++ | - | - | - | - | + | - |
| 9 | | | - | - | - | ++ | - | ++ | - | - | - | - | + | - |
| 10 | | | - | - | - | - | + | - | - | - | - | ++ | - | + |
| 11 | | | - | - | - | - | - | + | - | - | + | + | - | - |
| 12 | | | - | - | - | - | - | + | - | - | ++ | - | - | - |
| 13 | | | - | - | - | - | + | - | - | - | + | + | - | - |
| 14 | | | - | - | - | + | - | - | - | - | - | - | + | - |
| 15 | | | - | + | - | - | - | + | - | - | - | - | - | - |
| 16 | | | - | - | - | - | - | - | - | - | + | - | - | - |

[a] Each antibiogram profile reflects an MIC combination that was identified among the CF67 isolates for the antibiotics aztreonam and ceftazidime.

[b] MICs were grouped into the following susceptibility categories based on CLSI interpretive criteria:

Susceptible | Intermediate | Resistant (MIC at breakpoint) | Highly resistant (MIC at least 2-fold higher than breakpoint)

[c] Frequency is expressed as the number of isolates with the antibiogram per timepoint, where (-) was not observed, (+) a single isolate, (++) 2 isolates, (+++) 3 or more isolates

Table S3. Antimicrobial therapies administered to patient CF67 during the 350 day sampling period

| Therapy[a] | Delivery[c] | Time from first sample (days)[b] Start | Stop |
|---|---|---|---|
| Azithromycin | PO | -169 | 361 |
| Aztreonam | IV | 219 | 224 |
| | | 224 | 253 |
| | | 263 | 301 |
| | | 301 | 311 |
| | INH | 325 | 357 |
| Ceftazidime | IV | 170 | 185 |
| Chloramphenicol | IV | 0 | 14 |
| | | 231 | 233 |
| | | 306 | 311 |
| Ciprofloxacin | PO | 77 | 85 |
| | | 108 | 128 |
| | | 162 | 172 |
| | | 317 | 361 |
| Colistin | IV | 0 | 28 |
| | | 77 | 108 |
| | | 219 | 224 |
| | | 224 | 230 |
| | | 263 | 301 |
| | INH | -401 | 0 |
| | | 29 | 77 |
| | | 108 | 219 |
| | | 231 | 263 |
| Gentamicin | PO | 94 | 108 |
| | | 122 | 135 |
| Metronidazole | PO | 308 | 323 |
| | | 337 | 357 |
| Piperacillin-Tazobactam | IV | 15 | 28 |
| | | 85 | 108 |
| | | 233 | 253 |
| Tobramycin | IV | 301 | 305 |

[a] Clinical metadata includes all antimicrobial therapies prescribed during the 350 day study period

[b] Treatments are presented by the time of initiation (Start) and subsequent discontinuation (Stop) relative to the initial study specimen (day 0)
Negative values indicate that the treatment was initiated prior to our first study specimen

[c] Method of delivery is specified by the route of antimicrobial administration, whether oral (PO), parenteral (IV) or inhaled (INH)

Table S4. Chi square analysis of morphotype-phenotype relationships for continous phenotypes

| Phenotypes | | Morphotypes in highest abundance within CF67 *P. aeruginosa* population [% (n)] | | | | | | | | | | | |
|---|---|---|---|---|---|---|---|---|---|---|---|---|---|
| | | BMOS | | RMOS | | TMOS | | TMOSH | | WMOS | | WMTrSH | |
| | | Yes | No | Yes | No | Yes | No | Yes | No | Yes | No | Yes | No |
| Antimicrobial susceptibility | | | | | | | | | | | | | |
| Aztreonam MIC | Below breakpoint | - | 44.2 (99) | 33.3 (18) | 44.7 (81) | 48.9 (22) | 40.5 (77) | 47.8 (11) | 41.5 (88) | 39.0 (16) | 42.8 (83) | 6.7 (1) | 44.6 (98) |
| | At breakpoint | - | 14.7 (33) | 18.5 (10) | 12.7 (23) | 8.9 (4) | 15.3 (29) | 34.8 (8) | 11.8 (25) | 12.2 (5) | 14.4 (28) | 6.7 (1) | 14.6 (32) |
| | Above breakpoint | 100 (11) | 41.1 (92) | 48.2 (26) | 42.5 (77) | 42.2 (19) | 44.2 (84) | 17.4 (4) | 46.7 (99) | 48.8 (20) | 42.8 (83) | 86.7 (13) | 41.0 (90) |
| | n | 11 | 224 | 54 | 181 | 45 | 190 | 23 | 212 | 41 | 194 | 15 | 220 |
| | Comparisons | Above breakpoint Y vs N | | ns | | ns | | Above breakpoint Y vs N | | ns | | Above breakpoint Y vs N | |
| | *p* value[b] | *p* = 0.006 | | *p* = 0.2745 | | *p* = 0.4297 | | *p* = 0.0024 | | *p* = 0.7742 | | *p* = 0.0023 | |
| Ceftazidime MIC | Below breakpoint | 9.1 (1) | 47.3 (106) | 38.9 (21) | 47.5 (86) | 57.8 (26) | 42.6 (81) | 43.4 (10) | 45.8 (97) | 39.0 (16) | 46.9 (91) | 33.3 (5) | 46.4 (102) |
| | At breakpoint | - | 15.6 (35) | 14.8 (8) | 14.9 (27) | 8.9 (4) | 16.3 (31) | 1.74 (1) | 16.0 (34) | 9.8 (4) | 16.0 (31) | 60.0 (9) | 11.8 (26) |
| | Above breakpoint | 90.9 (10) | 37.1 (83) | 46.3 (25) | 37.6 (68) | 33.4 (15) | 41.1 (78) | 52.2 (12) | 38.2 (81) | 51.2 (21) | 37.1 (72) | 6.7 (1) | 41.8 (92) |
| | n | 11 | 224 | 54 | 181 | 45 | 190 | 23 | 212 | 41 | 194 | 15 | 220 |
| | Comparisons | Above breakpoint Y vs N | | ns | | ns | | ns | | ns | | At breakpoint Y vs N | |
| | *p* value[b] | *p* = 0.0017 | | *p* = 0.4770 | | *p* = 0.1550 | | *p* = 0.2288 | | *p* = 0.2183 | | *p* < 0.0001 | |
| Ciprofloxacin MIC | Below breakpoint | - | 42.4 (95) | 42.3 (23) | 39.8 (72) | 46.7 (21) | 39.0 (74) | 52.2 (12) | 39.2 (83) | 31.7 (13) | 42.3 (82) | 6.7 (1) | 42.7 (94) |
| | At breakpoint | 100 (11) | 56.7 (127) | 57.4 (31) | 59.1 (107) | 51.1 (23) | 60.5 (115) | 47.8 (11) | 59.9 (127) | 68.3 (28) | 56.7 (110) | 93.3 (14) | 56.4 (124) |
| | Above breakpoint | - | 0.9 (2) | - | 1.1 (2) | 2.2 (1) | 0.5 (1) | - | 0.9 (2) | - | 1.0 (2) | - | 0.9 (2) |
| | n | 11 | 224 | 54 | 181 | 45 | 190 | 23 | 212 | 41 | 194 | 15 | 220 |
| | Comparisons | At breakpoint Y vs N | | ns | | ns | | ns | | ns | | At breakpoint Y vs N | |
| | *p* value[b] | *p* = 0.0173 | | *p* = 0.7051 | | *p* = 0.3143 | | *p* = 0.4486 | | *p* = 0.3446 | | *p* = 0.0190 | |
| Secreted virulence factors and metabolism | | | | | | | | | | | | | |
| Amino acid auxotrophy | Prototrophy | 27.3 (3) | 72.8 (163) | 18.5 (10) | 29.8 (54) | 35.6 (16) | 74.7 (142) | 39.1 (9) | 74.1 (157) | 24.4 (10) | 27.8 (54) | 46.7 (7) | 25.9 (57) |
| | Auxotrophy | 72.7 (8) | 27.2 (61) | 81.5 (44) | 18.5 (10) | 64.4 (29) | 25.3 (48) | 60.9 (14) | 25.9 (55) | 75.6 (31) | 72.2 (140) | 53.3 (8) | 74.1 (163) |
| | n | 11 | 224 | 54 | 181 | 45 | 190 | 23 | 212 | 41 | 194 | 15 | 220 |
| | Comparisons | ns | | ns | | ns | | ns | | ns | | ns | |
| | *p* value[b] | *p* = 0.9976 | | *p* = 0.1011 | | *p* = 0.1631 | | *p* = 0.1772 | | *p* = 0.6525 | | *p* = 0.0806 | |
| EPS production | None | - | 8.9 (20) | - | 11.0 (20) | - | 10.5 (20) | 4.3 (1) | 9.0 (19) | 2.4 (1) | 9.8 (19) | - | 9.1 (20) |
| | Light to moderate | 81.8 (9) | 32.14 (72) | 24.1 (13) | 37.6 (68) | 20.0 (9) | 37.9 (72) | 39.1 (9) | 34.0 (72) | 31.7 (13) | 35.1 (68) | 80.0 (12) | 31.4 (69) |
| | Heavy | 18.2 (2) | 58.9 (132) | 75.9 (41) | 51.4 (93) | 80.0 (36) | 51.6 (98) | 56.5 (13) | 57.1 (121) | 65.9 (27) | 55.1 (107) | 20.0 (3) | 59.5 (131) |
| | n | 11 | 224 | 54 | 181 | 45 | 190 | 23 | 212 | 41 | 194 | 15 | 220 |
| | Comparisons | Heavy Y vs N | | Heavy Y vs N | | Heavy Y vs N | | ns | | ns | | Light to moderate Y vs N | |
| | *p* value[b] | *p* = 0.0031 | | *p* = 0.0019 | | *p* = 0.0013 | | *p* = 0.7114 | | *p* = 0.2299 | | *p* = 0.0006 | |
| Surface sheen[a] | Present | 27.3 (3) | 67.4 (151) | 83.3 (45) | 60.2 (109) | 82.2 (37) | 61.6 (117) | 73.9 (17) | 64.6 (137) | 58.5 (24) | 67.0 (130) | 33.3 (5) | 67.7 (149) |
| | Absent | 72.7 (8) | 27.3 (3) | 16.7 (9) | 39.8 (72) | 17.8 (8) | 38.4 (73) | 26.1 (6) | 35.4 (75) | 41.5 (17) | 33.0 (64) | 66.7 (10) | 32.3 (71) |
| | n | 11 | 224 | 54 | 181 | 45 | 190 | 23 | 212 | 41 | 194 | 15 | 220 |
| | Comparisons | Present Y vs N | | Present Y vs N | | Present Y vs N | | ns | | ns | | Present Y vs N | |
| | *p* value[b] | *p* = 0.0062 | | *p* = 0.0017 | | *p* = 0.0088 | | *p* = 0.3723 | | *p* = 0.2996 | | *p* = 0.0067 | |
| Protease production | Present | 9.1 (1) | 12.5 (28) | 20.4 (11) | 9.9 (18) | 15.6 (7) | 11.6 (22) | - | 13.7 (29) | 2.4 (1) | 14.4 (28) | 6.7 (1) | 12.7 (28) |
| | Absent | 90.9 (10) | 87.5 (196) | 79.6 (43) | 90.1 (163) | 84.4 (38) | 88.4 (168) | 100.0 (23) | 86.3 (183) | 97.6 (40) | 85.6 (166) | 93.3 (14) | 87.3 (192) |
| | n | 11 | 224 | 54 | 181 | 45 | 190 | 23 | 212 | 41 | 194 | 15 | 220 |
| | Comparisons | ns | | Present Y vs N | | ns | | Absent Y vs N | | Present Y vs N | | ns | |
| | *p* value[b] | *p* = 0.7372 | | *p* = 0.0409 | | *p* = 0.4658 | | *p* = 0.0582 | | *p* = 0.0339 | | *p* = 0.4899 | |
| Pyocyanin production | None | 90.9 (10) | 39.7 (89) | 44.4 (24) | 41.4 (75) | 55.6 (25) | 38.5 (74) | 13.0 (3) | 45.3 (96) | 53.7 (22) | 39.7 (77) | 13.3 (2) | 44.1 (97) |
| | Moderate | 9.1 (1) | 42.0 (94) | 37.1 (20) | 41.4 (75) | 28.9 (13) | 43.2 (82) | 52.2 (12) | 39.1 (83) | 41.5 (17) | 40.2 (78) | 86.7 (13) | 37.3 (82) |
| | High | - | 18.3 (41) | 18.5 (10) | 17.1 (31) | 15.6 (7) | 17.9 (34) | 34.8 (8) | 15.6 (33) | 4.9 (2) | 20.1 (39) | - | 18.6 (41) |
| | n | 11 | 224 | 54 | 181 | 45 | 190 | 23 | 212 | 41 | 194 | 15 | 220 |
| | Comparisons | High Y vs N | | ns | | ns | | High Y vs N | | High Y vs N | | High Y vs N | |
| | *p* value[b] | *p* = 0.0035 | | *p* = 0.8459 | | *p* = 0.1148 | | *p* = 0.0056 | | *p* = 0.0479 | | *p* = 0.0007 | |
| Siderophore production | None | 27.3 (3) | 46.0 (103) | 53.7 (29) | 42.5 (77) | 66.7 (30) | 40.0 (76) | 52.2 (12) | 44.3 (94) | 48.8 (20) | 44.3 (86) | 13.3 (2) | 42.3 (104) |
| | Moderate | 63.6 (7) | 42.4 (95) | 31.5 (17) | 49.7 (85) | 17.8 (8) | 49.5 (94) | 47.8 (11) | 42.9 (91) | 39.0 (16) | 44.3 (86) | 80.0 (12) | 40.9 (90) |
| | High | 9.1 (1) | 11.6 (26) | 14.8 (8) | 10.5 (19) | 15.6 (7) | 10.5 (20) | - | 12.7 (27) | 12.2 (5) | 11.3 (22) | 6.7 (1) | 11.8 (26) |
| | n | 11 | 224 | 54 | 181 | 45 | 190 | 23 | 212 | 41 | 194 | 15 | 220 |
| | Comparisons | ns | | ns | | High Y vs N | | ns | | ns | | Moderate Y vs N | |
| | *p* value[b] | *p* = 0.3753 | | *p* = 0.1275 | | *p* = 0.0006 | | *p* = 0.1895 | | *p* = 0.8230 | | *p* = 0.0119 | |

[a] Identified in mucoid isolates as small aggregated particles with an iridescent sheen on the alginate layer, while the entire colony of non-mucoid isolates was iridescent

[b] Only *p* values of ≤0.004 were considered significant at 95% confidence (Bonferroni corrected)

Table S5. Morphotype-phenotype relationships for categorical phenotypes

| Phenotypes | | Morphotypes of highest abundance in CF67 sputum[a] | | | | | | | | | | | |
|---|---|---|---|---|---|---|---|---|---|---|---|---|---|
| | | BMOS | | RMOS | | TMOS | | TMOSH | | WMOS | | WMTrSH | |
| | | Yes | No | Yes | No | Yes | No | Yes | No | Yes | No | Yes | No |
| Motility-associated | | | | | | | | | | | | | |
| Biofilm formation | Mean ($OD_{550}$) | 0.752 | 1.18 | 1.13 | 1.17 | 1.26 | 1.14 | 1.13 | 1.17 | 1.15 | 1.16 | 0.372 | 1.22 |
| | Std Error | 0.21 | 0.063 | 0.122 | 0.07 | 0.138 | 0.068 | 0.2 | 0.064 | 0.136 | 0.068 | 0.0852 | 0.0631 |
| | df | 12 | | 91 | | 67 | | 27 | | 62 | | 33 | |
| | t value | 2.0 | | 0.33 | | -0.75 | | 0.18 | | 0.12 | | 7.97 | |
| | Comparisons | ns | | ns | | ns | | ns | | ns | | WMTrSH Y vs N | |
| | *p* value[b] | $p = 0.07$ | | $p = 0.741$ | | $p = 0.454$ | | $p = 0.856$ | | $p = 0.905$ | | $p < 0.0001$ | |
| Swarming | Mean (mm) | 10.6 | 11.2 | 11.8 | 10.9 | 10.6 | 11.3 | 12.7 | 11.0 | 11.4 | 11.1 | 11.0 | 11.14 |
| | Std Error | 0.705 | 0.311 | 0.625 | 0.339 | 0.727 | 0.327 | 0.613 | 0.322 | 0.871 | 0.312 | 0.632 | 0.316 |
| | df | 14 | | 86 | | 63 | | 35 | | 51 | | 22 | |
| | t value | 0.71 | | -1.20 | | 0.87 | | -2.45 | | -0.38 | | 0.21 | |
| | Comparisons | ns | | ns | | ns | | ns | | ns | | ns | |
| | *p* value[b] | $p = 0.488$ | | $p = 0.234$ | | $p = 0.388$ | | $p = 0.019$ | | $p = 0.704$ | | $p = 0.833$ | |
| Swimming | Mean (mm) | 2.04 | 3.77 | 3.75 | 3.67 | 4.64 | 3.47 | 1.31 | 4.0 | 1.62 | 4.13 | 2.70 | 3.76 |
| | Std Error | 0.548 | 0.423 | 0.733 | 0.478 | 1.05 | 0.434 | 0.363 | 0.443 | 0.691 | 0.462 | 1.54 | 0.420 |
| | df | 25 | | 102 | | 60 | | 111 | | 80 | | 16 | |
| | t value | 2.49 | | -0.09 | | -1.03 | | 4.61 | | 3.01 | | 0.66 | |
| | Comparisons | ns | | ns | | ns | | TMOSH Y vs N | | WMOS Y vs N | | ns | |
| | *p* value[b] | $p = 0.02$ | | $p = 0.928$ | | $p = 0.308$ | | $p < 0.0001$ | | $p = 0.0035$ | | $p = 0.516$ | |
| Twitching | Mean (mm) | 2.53 | 2.41 | 2.62 | 2.36 | 2.00 | 2.52 | 1.02 | 2.57 | 2.11 | 2.48 | 2.43 | 2.42 |
| | Std Error | 0.178 | 0.189 | 0.242 | 0.222 | 0.489 | 0.19 | 0.301 | 0.194 | 0.37 | 0.204 | 1.16 | 0.176 |
| | df | 42 | | 149 | | 58 | | 43 | | 68 | | 15 | |
| | t value | -0.45 | | -0.78 | | 0.97 | | 4.32 | | 0.9 | | -0.01 | |
| | Comparisons | ns | | ns | | ns | | TMOSH Y vs N | | ns | | ns | |
| | *p* value[b] | $p = 0.654$ | | $p = 0.437$ | | $p = 0.338$ | | $p < 0.0001$ | | $p = 0.372$ | | $p = 0.99$ | |

[a] Differences in phenotypic responses were tested between a given colony morphotype (with n≥10) and the remaining isolates which were not of that morphotype

[b] Only *p* values of ≤0.004 considered significant at 95% confidence (Bonferroni corrected)
